# Large‐scale production of extracellular vesicles: Report on the “massivEVs” ISEV workshop

**DOI:** 10.1002/jex2.63

**Published:** 2022-10-25

**Authors:** Lucia Paolini, Marta Monguió‐Tortajada, Marta Costa, Fabio Antenucci, Mario Barilani, Marta Clos‐Sansalvador, André Cronemberger Andrade, Tom A. P. Driedonks, Sara Giancaterino, Stephanie M. Kronstadt, Rachel R. Mizenko, Muhammad Nawaz, Xabier Osteikoetxea, Carla Pereira, Surya Shrivastava, Anders Toftegaard Boysen, Simonides Immanuel van de Wakker, Martijn J. C. van Herwijnen, Xiaoqin Wang, Dionysios C. Watson, Mario Gimona, Maria Kaparakis‐Liaskos, Konstantin Konstantinov, Sai Kiang Lim, Nicole Meisner‐Kober, Michiel Stork, Peter Nejsum, Annalisa Radeghieri, Eva Rohde, Nicolas Touzet, Marca H. M. Wauben, Kenneth W. Witwer, Antonella Bongiovanni, Paolo Bergese

**Affiliations:** ^1^ Department of Molecular and Translational Medicine University of Brescia Brescia Italy; ^2^ Center for Colloid and Surface Science (CSGI) Department of Chemistry University of Florence Sesto Fiorentino (FI) Italy; ^3^ Department of Medical and Surgical Specialties Radiological Sciences and Public Health University of Brescia Brescia Italy; ^4^ CREC Research Program Health Science Research Institute Germans Trias i Pujol (IGTP) Badalona Spain; ^5^ Heart Institute (iCor) Cardiology Department Germans Trias i Pujol University Hospital Badalona Spain; ^6^ iBET Instituto de Biologia Experimental e Tecnológica Oeiras Portugal; ^7^ Instituto de Tecnologia Química e Biológica António Xavier Universidade Nova de Lisboa Oeiras Portugal; ^8^ Department of Veterinary and Animal Sciences University of Copenhagen Copenhagen Denmark; ^9^ Laboratory of Regenerative Medicine ‒ Cell Factory Department of Transfusion Medicine and Hematology Fondazione IRCCS Ca' Granda Ospedale Maggiore Policlinico Milano Italy; ^10^ REMAR‐IGTP Group Germans Trias i Pujol Research Institute (IGTP) & Nephrology Department University Hospital Germans Trias i Pujol (HUGTiP) Badalona (Barcelona) Catalonia Spain; ^11^ Department of Cell Biology Physiology and Immunology Universitat Autònoma de Barcelona (UAB) Bellaterra Spain; ^12^ Experimental and Clinical Cell Therapy Institute (ExCT) Spinal Cord Injury and Tissue Regeneration Center Paracelsus Medical University Salzburg Austria; ^13^ CDL Research University Medical Center Utrecht Utrecht University Utrecht The Netherlands; ^14^ Department of Molecular and Comparative Pathology Johns Hopkins School of Medicine Baltimore Maryland USA; ^15^ Department of Civil Chemical Environmental and Materials Engineering Alma Mater Studiorum University of Bologna Bologna Italy; ^16^ Fischell Department of Bioengineering University of Maryland College Park Maryland USA; ^17^ Department of Biomedical Engineering University of California Davis California USA; ^18^ Department of Rheumatology and Inflammation Research Institute of Medicine Sahlgrenska Academy University of Gothenburg Gothenburg Sweden; ^19^ HCEMM‐SU Extracellular Vesicles Research Group Budapest Hungary; ^20^ Department of Genetics Cell‐ and Immunobiology Semmelweis University Budapest Hungary; ^21^ Exogenus Therapeutics S.A. Cantanhede Portugal; ^22^ Center for Gene Therapy City of Hope Beckman Research Institute and Hematological Malignancy and Stem Cell Transplantation Institute at the City of Hope Duarte California USA; ^23^ Department of Clinical Medicine Aarhus University Aarhus Denmark; ^24^ Department of Infectious Diseases Aarhus University Hospital Aarhus Denmark; ^25^ Department of Cardiology Experimental Cardiology Laboratory University Medical Center Utrecht Utrecht University Utrecht The Netherlands; ^26^ Department of Biomolecular Health Sciences Faculty of Veterinary Medicine Utrecht University Utrecht Netherlands; ^27^ Discovery Biology Discovery Sciences BioPharmaceuticals R&D AstraZeneca Gothenburg Sweden; ^28^ University Hospitals Cleveland Medical Center Cleveland Ohio USA; ^29^ Case Western Reserve University Cleveland Ohio USA; ^30^ Cleveland Clinic Cleveland Ohio USA; ^31^ GMP Unit Spinal Cord Injury & Tissue Regeneration Centre Salzburg (SCI‐TReCS) Paracelsus Medical University Salzburg Austria; ^32^ Research Program “Nanovesicular Therapies” Paracelsus Medical University Salzburg Austria; ^33^ Transfer Centre for Extracellular Vesicle Theralytic Technologies (EV‐TT) Salzburg Austria; ^34^ Department of Microbiology, Anatomy, Physiology and Pharmacology La Trobe University Melbourne Victoria Australia; ^35^ La Trobe Research Centre for Extracellular vesicles La Trobe University Melbourne Victoria Australia; ^36^ Codiak BioSciences Cambridge Massachusetts USA; ^37^ Exosome and Secreted Nano‐vesicle Institute of Molecular & Cell Biology Singapore Singapore; ^38^ Department of Biosciences and Medical Biology Paris‐Lodron University Salzburg Hellbrunnerstrasse 34 Salzburg Austria; ^39^ Intravacc Bilthoven The Netherlands; ^40^ Department of Transfusion Medicine University Hospital Salzburger Landeskliniken GesmbH (SALK) of Paracelsus Medical University Salzburg Austria; ^41^ Centre for Environmental Research Innovation and Sustainability Institute of Technology Sligo Sligo Ireland; ^42^ Department of Neurology Johns Hopkins University School of Medicine Baltimore Maryland USA; ^43^ Cell‐Tech HUB and Institute for Research and Biomedical Innovation National Research Council of Italy (CNR) Palermo Italy

**Keywords:** EV‐based products, extracellular vesicles, ISEV workshop, large‐scale production, manufacturing, processing, regulatory issues

## Abstract

Extracellular vesicles (EVs) large‐scale production is a crucial point for the translation of EVs from discovery to application of EV‐based products. In October 2021, the International Society for Extracellular Vesicles (ISEV), along with support by the FET‐OPEN projects, “The Extracellular Vesicle Foundry” (evFOUNDRY) and “Extracellular vesicles from a natural source for tailor‐made nanomaterials” (VES4US), organized a workshop entitled “massivEVs” to discuss the potential challenges for translation of EV‐based products. This report gives an overview of the topics discussed during “massivEVs”, the most important points raised, and the points of consensus reached after discussion among academia and industry representatives. Overall, the review of the existing EV manufacturing, upscaling challenges and directions for their resolution highlighted in the workshop painted an optimistic future for the expanding EV field.

## 1 INTRODUCTION

Extracellular vesicles (EVs) are biological nanoparticles delimited by a lipid bilayer (nominal size. ranging from 30 to 1000 nm) that are released by cells to act as intercellular signalling mediators in both physiological and pathological conditions (Busatto et al., 2019; Yáñez‐Mó et al., 2015). This process is conserved throughout evolution from bacteria to animals (humans) and plants (Adamo et al., 2021; Gill et al., 2018; Van Niel et al., 2018). As such, EVs are reshaping our perspective on life sciences, environment and public health.

The International Society for Extracellular Vesicles (ISEV) was founded in 2012, and its mission is to advance EV research globally. ISEV connects more than 2000 total members and organizes events including annual meetings, symposia, virtual courses (Massive Open Online Course, MOOC, I and II), journal clubs (Extracellular Vesicle Club), task force meetings organized by the “Rigor and Standardization Subcommittee”, and specialized workshops.

The ISEV workshops are among the most effective events to enhance the conception, and sharing of new perspectives in the EV field. The format for workshops was created by the ISEV board and started in 2012. Since then, sixteen workshops have been organized on different subjects: from basic research on EVs (isolation and characterization of EVs, EV RNA content (Hill et al., 2013; Mateescu et al., 2017), membrane analyses (Russell et al., 2019), research standardization, EV imaging), to EV involvement in cross‐organism communication (Soares et al., 2017), diet and environment, and to more clinically oriented topics, such as EVs in immunology, infectious diseases, theranostics and therapeutic applications (Clayton et al., 2018; Reiner et al., 2017; Soekmadji et al., 2020; Witwer et al., 2019) (Table 1).

**TABLE 1:** ISEV workshops organized from 2012 to 2022

Workshop	City	Country	Year
evRNA analysis and bioinformatics (Hill et al., 2013)	New York	USA	2012
Isolation and characterization of extracellular vesicles	Budapest	Hungary	2013
EV Therapeutics (Reiner et al., 2017) (ISEV‐SOCRATES joint)	Singapore	Singapore	2015
EV‐associated RNA: Is there a purpose? (Mateescu et al., 2017)	Utrecht	The Netherlands	2015
Cross‐organism communication by extracellular vesicles: Hosts, microbes, parasites (Soares et al., 2017)	Sao Paulo	Brazil	2016
Diet, environment and extracellular vesicles	Melbourne	Australia	2017
Extracellular vesicles as biomarkers of disease (Clayton et al., 2018)	Birmingham	United Kingdom	2017
Membranes and EVs workshop (Russell et al., 2019)	Baltimore	United States	2018
EV‐based clinical theranostics (Soekmadji et al., 2020)	Guangzhou	China	2018
Develop a standardized defining criteria for human MSC small EVs (Witwer et al., 2019) (ISEV‐SOCRATES joint)	Singapore	Singapore	2018
Open, reproducible and standardized EV research	Ghent	Belgium	2019
EVs in immunology	Buenos Aires	Argentina	2020
EV imaging in vivo	Virtual	Virtual	2020
Infectious diseases meeting	Virtual	Virtual	2021
MassivEVs	Desenzano del Garda	Italy/Virtual	2021
Blood EVs	Helsinki	Finland	2022

“MassivEVs” is a workshop dedicated to issues pertaining to large‐scale production of EVs, was held both “in person” (in Desenzano del Garda, Italy) and virtually on the 28^th^ and 29^th^ of October 2021. The event was co‐organized with two H2020‐FET‐OPEN projects, “The Extracellular Vesicle Foundry” (evFOUNDRY ‐ www.evfoundry.eu) and “Extracellular vesicles from a natural source for tailor‐made nanomaterials” (VES4US ‐ www.ves4us.eu), with the support of the University of Brescia and the Italian Society for Extracellular Vesicles (EVIta). This report presents an overview of the aims and structure of the workshop (Box 1), highlighting the viewpoints, challenges, new perspectives and points of consensus reached after discussion by the “massivEVs” participants both from academia and industry representatives.

## 2 “MASSIVEVS” WORKSHOP FEATURES

The goal of the “massivEVs” workshop was to address and channel efforts towards large‐scale (from lab‐ to preindustrial/industrial‐scale) EV production and pharmaceutical manufacturing. Themes ranged from technologies and equipment for EV manufacturing, upscaling issues, different starting sources and applications, process development in compliance to good manufacturing practices (GMPs), product validation and regulatory problems. While research in EV biology has progressed exponentially, major gaps remain in our understanding of EV biogenesis, release, target cell uptake and function (Zipkin, 2020). Despite this, the immense potential utility of EVs has attracted commercial interest such as the setting up of EV biotechnology companies and the pivoting of business focus to EVs by contract manufacturing organizations, and pharmaceutical companies (Silva et al., 2021). This development heightens the importance of academic and industrial research collaboration in establishing scientifically robust validation across the entire value chain of EV manufacturing that includes bioprocessing scalability, product efficacy, reproducibility, stability, safety, time and costs of production and compliance with regulatory oversights. These matters were identified by the IOC as potential bottlenecks that need to be efficiently and urgently addressed to enable the translation of EVs from discovery to broad applications, that is, human or veterinary clinical, nutraceutical, or cosmeceutical applications.

**Box 1‐ MassivEV Workshop Overview**

**Topics**: Four emerging and compelling points covering the most important issues related to the workshop (ws) theme.

**International Organizing committee (IOC)**: scientists who proposed the ws theme together with the ISEV board. Responsible for ws organization.

**Attendees**: ∼ 50 attendees selected on the basis of research area, expertise, abstracts outlining key interests, motivation statements.

**Introductory lectures**: two presentations for each topic by experts in the field. Invited speakers give an overview on issues and provide hints for further consideration.

**Selected oral presentations**: two presentations for each topic by attendees. They stimulate the scientific debate, bring in “state‐of‐the‐art” research and open questions.

**Round‐tables**: active discussions moderated by IOC and invited speakers to examine issues raised during talks. Attendees express their points of view and share their experience.

**Poster session**: it allows more participants to present data, enhance cross‐fertilization, collaborations and inputs among attendees with different expertise.

This report aims to give a snapshot of the workshop and post‐workshop activities and to summarize the major outcomes. A second “massivEVs product”, elaborated by all attendees, aiming at translating the workshop proceedings into a guideline/perspective paper is in preparation.

### 2.1 Participants

**FIGURE 1:**
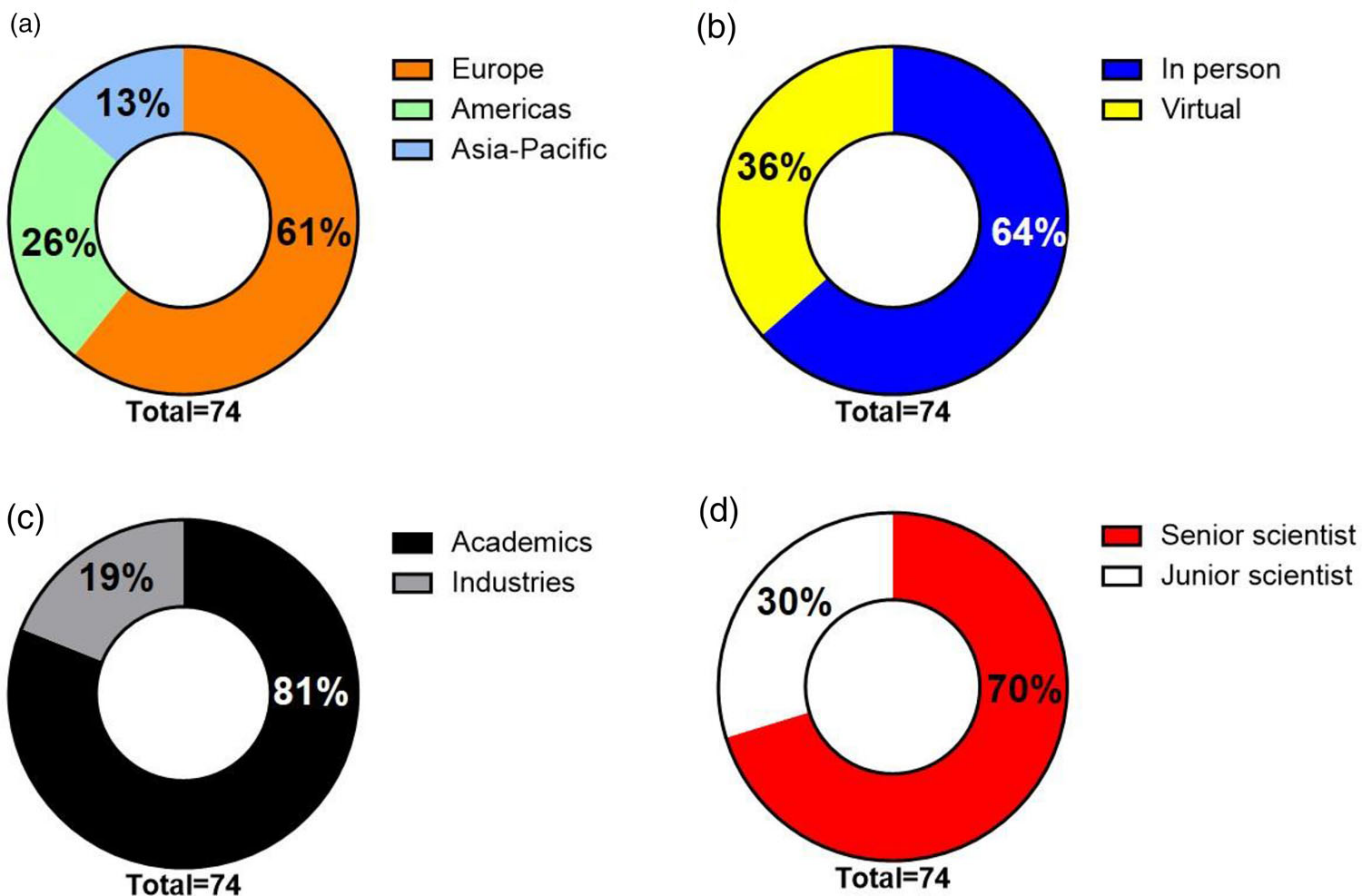
Graphs of participant sub‐division and percentage of each group on total of 74 attendees (comprising IOC, introductory speakers and scientists with selected abstracts): attendees depicted according to (a) ISEV geographical chapters represented; (b) in person or virtual presence; (c) affiliation to academics or industries; (d) senior (more than 4 years from Medicinae Doctor‐ MD‐, Philosophiae Doctor ‐ PhD‐ or equivalent degree) or junior (MD, PhD or equivalent degree not yet obtained or obtained within the previous 4 years) scientists

More than 100 applications were submitted for participation in the “massivEVs” workshop. Fifty‐eight scientists were selected by the IOC to attend the workshop. From this group, 13 were selected for oral presentations. ISEV geographical chapter representation was as follows (Figure 1a): Europe (61%), North and South America (Americas, 26%), and Asia and Oceania (Asia‐Pacific, 13%). Due to the COVID‐19 pandemic and related travel restrictions, the IOC decided to enable remote participation for those individuals who were not able to participate in person. Even so, more than 60% of selected participants were present “in person”, allowing effective interaction (Figure 1b). Almost 20% of the attendees were representatives of biotech companies, indicating that the topics discussed in the workshop have a “broad‐spectrum” interest both for academic and industry members (Figure 1c). Both senior and junior scientists were present (Figure 1d).

### 2.2 Definition of terms

In the course of the workshop, specific key terms typically used in pharmaceutical development were discussed. These keywords are part of the technical terminology established in pharmaceutical disciplines, manufacturing and risk management. The most used during the workshop are explained in Box 2.

**Box 2 ‐ Quality by design (QbD).**

QbD is a systematic approach to product development that aims to ensure high quality of products.^1b^ QbD incorporates quality aspects into the manufacturing process instead of applying empirical quality testing to the end products. It emphasizes understanding of how critical process parameters in defined ranges affect final product quality. This approach combines scientific expertise with risk management tools.

**Safety by design**. Manufacturers must perform proactive risk assessments when developing drug products to integrate safety into the entire manufacturing process of a drug product. Measurable, critical safety^2b^ characteristics of the pharmaceutical product should be identified and monitored accordingly.

**Upstream and downstream processing**. Upstream processing refers to the initial steps of bioprocessing to produce active pharmaceutical ingredients,^3b^ in this specific case EVs. Upstream processing comprises all actions to produce large amounts of EVs. Downstream processing refers to separation of EVs from the complex mixture of the starting source^3b^ (cells, host cell proteins, cell debris, nutrients and waste materials) by purification steps.

^1b^Lipsitz et al. Quality Cell Therapy Manufacturing by Design. *Nat Biotechnol*. 2016, *34* (4), 393–400. https://doi.org/10.1038/nbt.3525

^2b^ https://www.gmp‐compliance.org/gmp‐news/new‐fda‐guidance‐on‐safety‐by‐design

^3b^ https://www.bioxcellence.com/our‐business/upstream‐downstream‐processing

### 2.3 Topics in focus

IOC selected four topics for round‐table discussions during the “massivEVs” workshop:

1. Massive production from human sources and applications (including therapeutics, nutraceutics, cosmetics, EV‐based nanotechnology);
2. Massive production from other (than) human sources and applications;
3. Upstream and downstream technologies and process upscaling;
4. Validation, standardization and regulatory issues.

**FIGURE 2:**
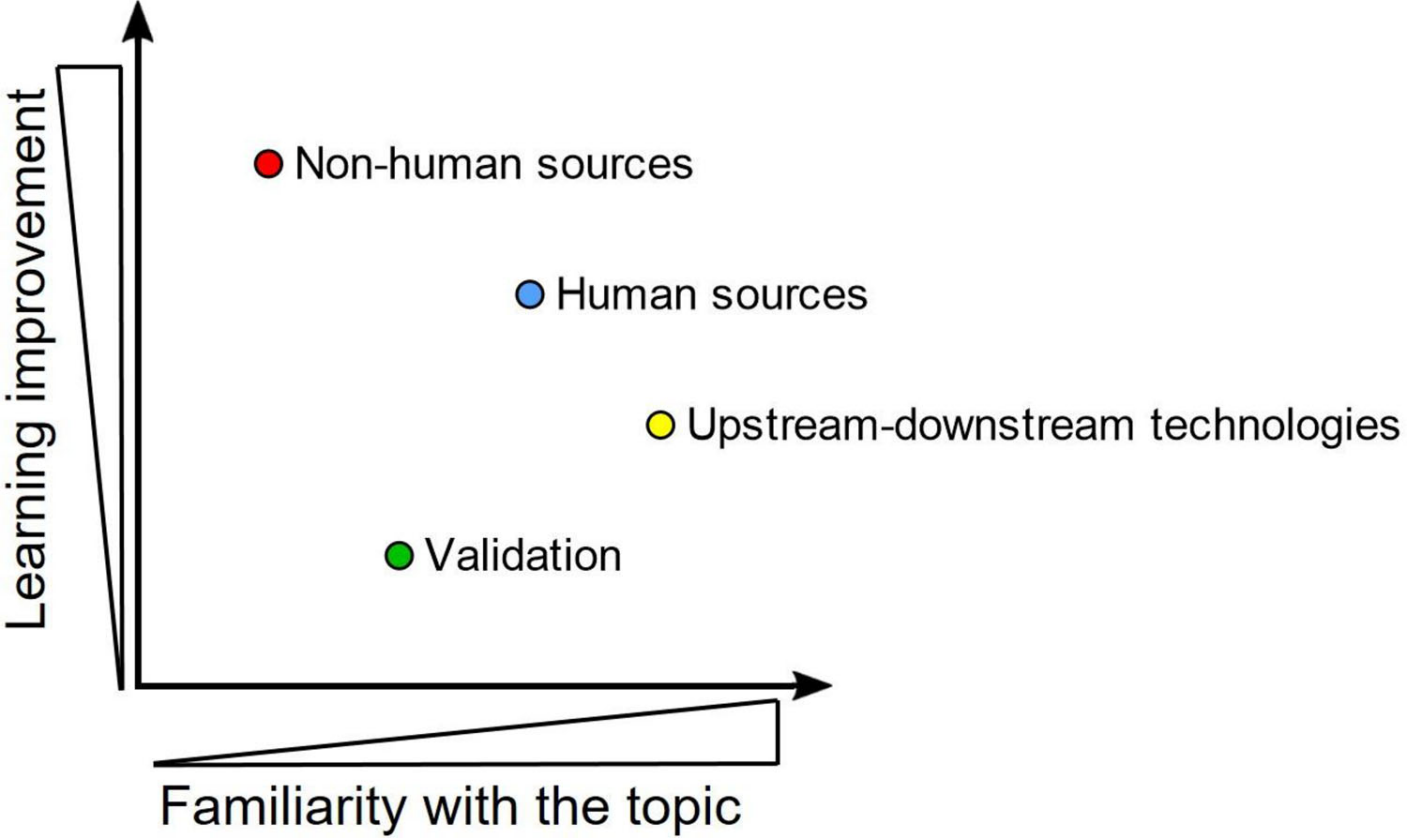
Ranking of familiarity with the topics (x‐axis) and level of learning improvement (y‐axis) among the “massivEVs” attendees. Topics were: (1) Massive production from human sources and applications (including therapeutics, nutraceutics, cosmetics, EV based nanotechnology), “Human sources” ‐ blue dot; (2) Massive production from other (than human) sources and applications, “Non‐human sources” ‐ red dot; (3) Upstream and downstream technologies and process upscaling, “Upstream‐downstream technologies” ‐ yellow dot; (4) Validation, standardization and regulatory issues, “Validation” ‐ green dot

In the post‐workshop survey, organizers asked participants to rank the topics according to their expertise from “most” to “least expertise”. The feedback of participants ranked “Upstream and downstream technologies and process upscaling” (topic 3) followed by “Massive production from human sources and applications” (topic 2) as those fields with which they felt most familiar. Fewer participants professed expertise in the fields of “Massive production from other (than) human sources and applications” (topic 2) and “Validation, standardization and regulatory issues” (topic 4) (Figure 2). The majority of attendees were aware of recent advances in scaling‐up and scaling‐out techniques for EV production (Grangier et al., 2021), including innovative bioreactors, in methods for improvement of upstream EV yield (Staubach et al., 2021) and in new technologies for scalable, cost‐effective and high‐throughput EV separation processes such as anion exchange chromatography (Heath et al., 2018) and tangential flow filtration (Paganini et al., 2019). The most discussed starting sources for EV separation were of human origin, such as body fluids (i.e., blood) or conditioned medium from mesenchymal stromal cells (MSC).

Attendees noted the highest learning improvement about “Massive production from other (than human) sources and applications” (topic 2) followed by topic 1, 3 and 4 (Figure 2). The non‐human sources of EVs raised interest among the participants, and this topic reached the highest score of “learning improvement” as some participants were not familiar with the fact that Evs can be separated from invertebrate culture medium, vegetables (e.g., lemon juice), microalgae, bovine milk, or bacterial supernatant.

Validation, standardization and regulatory issues (topic 4) remained the most unfamiliar subject for workshop attendees. This could be because regulatory guidance for EV‐based products is not yet well developed or standardized. In this respect, cross‐fertilization between academia, healthcare systems, industries and regulatory agencies is vital for successful development of EV‐based products.

## 3 KEY ISSUES RAISED DURING THE “MASSIVEVS” WORKSHOP

This section gives an overview of the most important and critical points raised during oral presentations and round‐table discussions of the four topics presented in the previous section. They highlight significant challenges and possible solutions to major hurdles in large‐scale production of EVs and their exploitation in different fields. Many of the challenges identified are shared themes between the four main topics of the workshop. Their discussion recurred during different moments of the event and cannot be investigated from a single point of view. This remarked that a multi‐disciplinary discussion is required to merge different backgrounds into a common vision.

### 3.1 EV‐based products: A balance among purity, safety and bioactivity

Depending on the source of EV and applications (nutraceuticals, cosmetics, or therapeutic fields) distinct levels of EV purity are likely to be required. EV preparations may contain different process‐related co‐isolates (the pharmaceutical technical term is “impurities”) and should be free of exogenous contaminants (“adventitious agents”). The latter include infectious microbes or microbial products (i.e., viruses, bacteria, prions, endotoxins) or toxic factors enriched during manufacturing and depending on starting material, disposables, or separation method used. EV co‐isolates can be: non‐EV particles, EV aggregates, soluble proteins and the EV “protein corona” (Hadjidemetriou & Kostarelos, 2017), a spontaneous self‐assembly layer of proteins that cover the surface of synthetic (Ducoli et al., 2022; Monopoli et al., 2011) and/or biogenic (Tóth et al., 2021) nanoparticles when interfacing with a biological matrix. These co‐isolates may be intrinsic from the starting material or may arise from the manufacturing process (Aminzadeh et al., 2021). Once “adventitious agents” are excluded, the acceptable purity levels of EV preparations must be defined to ensure safety, efficacy, and acceptable toxicity profiles and will greatly influence upstream and downstream processing, as well as quality controls. Since proposed measures of EV purity include ratios of particle counts to proteins, lipids, or other EV cargo (Osteikoetxea et al., 2015; Théry et al., 2018; Webber & Clayton, 2013), it is important to consider that the presence of co‐isolates can interfere with quantification of EVs and EV attributes. Attention to accuracy and reproducibility of these measurements is needed to make EV purity measurements more reliable. During the workshop, it was pointed out that EV purity may not correlate with EV functionality and co‐isolates in EV products could even contribute to the functions and stability of EV products (Gomes et al., 2022; Wolf et al., 2022). This depends on the starting material, downstream processing and mode of action of EVs (Mol et al., 2017). If co‐isolates are identified and cannot be removed without changing functionality, the recommendation is to characterize their mechanism of action in the EV‐based product and to evaluate reproducibility between batches (“batch‐to‐batch consistency”). Co‐isolates can be categorized as additionally needed components for an observed biological/therapeutic activity.

The purity range can be determined according to the range for which the relevant EV potency assays (defined for the specific intended use) complies with the target effect. Therefore, knowing the composition and heterogeneity of a starting sample would help to predict the type of expected co‐isolates that would be found in the EV product. Together with the target product profile and potency assays of the EV preparation, one can define the desired purity level and hence the most suitable purification method.

A similar approach can be applied to safety. One of the open questions raised during the workshop was: should we first show efficacy or safety? A simple answer that emerged was that there is no need to show safety of a (future) product if we do not observe an activity (potency/efficacy) in relevant models of an intended disease or condition.

In conclusion, translating successful EV‐related studies into EV‐based products requires careful consideration of purity, safety, and bioactivity profiles.

### 3.2 Defining quality metrics for EV‐products

It was generally agreed during the “massivEVs” workshop that quality metrics need to be defined for the analysis of EV products. These should be tailored for a specific EV product and its intended use, also considering the need to identify quantifiable parameters of EV structure and activity‐related assays. Even though the application of Quality by Design (QbD) to research‐grade and/or small‐scale EV production processes was deemed premature, a crucial accelerating step in early research would be the proactive consideration of QbD concepts. For example, the identification of potential Critical Quality Attributes (CQA) of a future EV‐based product that are measurable with robust, reliable and simple methods (see “Academia inspired by industry” section below). Those parameters should be relevant for the intended use and can be used for a reasonable Quality Control (QC) strategy of the novel EV product.

In conclusion, EV‐based products should be thoroughly characterized by a range of quantitative and descriptive assays, which can be reported/documented in a multi‐modal test matrix. Defining process control parameters is crucial to achieve and confirm batch‐to‐batch consistency as a precondition for reproducibility of functionality in *in vitro* and *in vivo* models. This was stressed to be particularly relevant as the EV field is currently at the intersection between basic discovery and preclinical/commercial development.

### 3.3 EV shelf life and stability

Issues about EV shelf life and stability, including sample logistics, shipment conditions, and optimal storage are often underestimated, but are key points for the commercialization and final use of EV‐based products (Le Saux et al., 2020; Van De Wakker et al., 2022; Zarovni et al., 2022). Since these themes have a wide‐ranging impact, partnerships between academia and industry are already working in this direction. A good example is represented by several scientific projects funded by the European Commission. Future Emerging Technologies (FET)‐OPEN and FET PROACTIVE projects like evFOUNDRY (https://cordis.europa.eu/project/id/801367/it), VES4US (https://cordis.europa.eu/project/id/801338/it) and BOW (https://cordis.europa.eu/project/id/952183/it) dedicated specific tasks to these topics in order to assess the best conditions for EV formulations for mid and long‐term storage.

### 3.4 Academia inspired by industry

To better manage sources of variability during EV manufacturing and, consequently, increase the safety and bioactivity of the EV products, learning from QbD and safety‐by‐design approaches has been proposed to the EV community. These approaches require an in‐depth understanding of process parameters but provide exciting opportunities opened up by the “design space” (Lipsitz et al., 2016; Zarovni et al., 2022), which allows for process modification within predefined ranges of critical parameters. Examples of process de‐risking include assessing viral clearance by validated analytical methods, for instance, in the cell bank that represents the starting material and on the use of safe starting materials such as through the use of chemically‐defined medium. Each decision for or against a specific process step or material depends on a prior risk assessment that considers all scientific knowledge available at this developmental stage.

For each EV processing step, it is important to determine the purpose of the step, its impact on the product, and associated risks for future use. Therefore, the “design space” should consider formulation and biological attributes, but should also include commercial translation to define an optimum operation range. For instance, both dose and demand should determine the EV manufacturing scale, and the shelf life of the product should also be evaluated to adjust the process scalability. However, while these aspects should ideally be considered during early stages of development of EV‐based products, it is essential that they do not impose unnecessary boundaries on the scientific process. Academic researchers are, in most cases, not equipped with adequate resources for early‐stage biopharmaceutical development, and it is a clear task of industrial research to solve late‐stage developmental challenges in product development. During this workshop, a transparent and fair exchange between academia and the biotechnological industry has been strongly encouraged as a strategy to boost interdisciplinary cross‐fertilization and most likely accelerate development of EV‐based products. In particular, it was recommended to assess the target production scale early in the process to optimize manufacturing design and reduce future costs by avoiding expensive changes in the production process.

### 3.5 The potential of non‐human sources

“MassivEVs” has contributed to acknowledge the great potential of non‐human sources for large‐scale production of EVs. Non‐human sources of EVs that were presented included: prokaryotes (Gram‐positive (Liu et al., 2018) and Gram‐negative (Schwechheimer & Kuehn, 2015) bacteria), bovine milk (Kleinjan et al., 2021), parasitic helminths (Marcilla et al., 2012) (i.e., excretory/secretory product from *Ascaris suum* (Borup et al., 2022)), plants (i.e., *Citrus limon* fruit, juice (Raimondo et al., 2015)), and protists (i.e., nanoalgosomes derived from the microalga *Tetraselmis chuii* (Adamo et al., 2021; Picciotto et al., 2021)). The high variability in the sources also reflects high versatility of these EVs. Researchers showed examples on how EVs from non‐human sources are more easily tunable in both upstream production and downstream modification in comparison to EVs from human sources. The potential fields in which these EVs can be exploited range from vaccines, therapeutics, drug delivery to nutraceutics and cosmetics. A particular example is the EVs from Gram‐negative bacteria known as outer membrane vesicles (OMVs). There are currently three *Neisseria meningitidis* serogroup B OMV‐based vaccines approved/marketed, the first two were developed in Cuba and in Norway in the 1980s (Petousis‐Harris, 2018). Lessons from the development of this advanced, vesicle‐based pharmaceutical can be exploited for various EV applications in the field. In fact, according to the discussions in the roundtable for topic 2, the perception on the most promising sources for exploitable EVs are firstly prokaryotes, and then, in order, vertebrates, plants, protists, and invertebrate animals.

However, some critical points still need to be addressed. Non‐human EVs can be immunogenic or allergenic depending on the administration route, dosage, and number/frequency of doses (long‐term, repeated administration (Gilmore et al., 2021; Kaparakis‐Liaskos & Ferrero, 2015)). In addition, care should be taken when performing studies examining the immunological effects of non‐human EVs, as variability in the methods used to generate and characterize these EVs will introduce experimental bias and has the potential to affect the immunological outcomes observed (Bitto et al., 2021; Liu et al., 2018). These factors must be carefully evaluated and tested in dedicated clinical trials, and will likely impact potential future applications. In addition, public opinion may have a profound impact on the acceptance of non‐human EV‐based products. The starting source and application can influence the perspective of non‐human EV usage for different purposes. For example, EVs derived from helminths are expected to be more accepted for therapeutic aims as opposed to nutraceutic applications, while, in cosmetics, EVs derived from plants or microalgae might be accepted by a wider number of customers than animal‐derived EV products.

### 3.6 Characteristics of upstream and downstream processing

At the workshop, clarifications were made to better distinguish upstream from downstream manufacturing as follows: “*Upstream* is in charge of producing more EVs, and *downstream* of losing less”.

As mainly highlighted in discussions related to topic 3, there was consensus that the line between the two processes is blurred, as they greatly influence each other. Thus, integrated upstream and downstream processes may be necessary to truly control EV production and increase EV yield. In this sense, it was acknowledged that different EV sources and applications may lead to great differences in manufacturing hurdles. For instance, researchers working with mammalian cell culture‐derived EVs may find main obstacles in the upstream processing, while those who study bacteria‐derived EVs or plants/blood/milk derived‐EVs would be mainly concerned with downstream limitations.

#### 3.6.1 Upstream processing

Workshop attendees portrayed a broad spectrum of the different EV sources and highlighted very different needs. The most promising mass production processes requiring simple upstream technologies shown in the meeting involved milk, blood and bacteria as EV sources. For researchers working with mammalian cells, especially MSCs as an EV source, upstream scale‐up (creating a more efficient process such as using a 3D‐bioreactor (Bellani et al., 2020)) was extensively discussed as a major hurdle in comparison with *out‐scaling* (multiplying the existing processes, such as using many conventional 2D‐culture flasks). Capable of industrial scale manufacturing, is the high‐density suspended culture of human cells grown in large bioreactors. On top of that, attendants shared different conditions to enhance EV production. For example, stimulation by shear stress (Patel et al., 2019) or ethanol (Patel et al., 2020) might enhance EV production, although the consistent function of these EVs, as well as impact on the producing cells, must be demonstrated throughout culture. Other approaches included host cell line engineering to increase productivity, which may be especially efficient for bacteria‐derived EVs or for immortalization of MSCs (Chen et al., 2011).

#### 3.6.2 Downstream processing

Downstream processing was considered by many attendants to be a challenging step with several limitations including to have minimal batch‐to‐batch variability, to provide cost‐effective solutions, to preserve EVs integrity and functions, to maximize product yield preferring high throughput technologies. The main hurdle is the lack of a gold standard separation process that can fit to different EV sources and applications. Nevertheless, after extensive discussion, there was a general agreement on the importance of finding the right balance between EV function, reproducibility, and purity (see “EV‐based products: a balance among purity, safety and bioactivity” section above).

Taking this into account, the most used downstream method appeared to be tangential flow filtration for large‐scale EV productions, which would then be analysed for function. Then, further purification would be applied if a more purified preparation would be needed by using size exclusion chromatography, affinity chromatography or ultracentrifugation (Théry et al., 2018).

### 3.7 Scientific argument and regulation

As mainly highlighted in discussions related to topic 4, regulatory issues should be considered early during the developmental process of a biopharmaceutical product. The EV topic is relatively new to regulatory experts; therefore, academia and industry stakeholders should closely interact with regulators to jointly develop and help define the rules and framework for EV‐based product development. Regulatory agencies are open for exchange and discussion: their role is not to limit scientist creativity, but to improve and inspire scientific progress, increase the quality of science and awareness on themes related to production processes that lead to a safe and efficacious EV product. Researchers should be aware of the process towards application of EV‐based products and consult experts from various disciplines, authorities and pertinent regulatory agencies from the very beginning of the research in order to greatly accelerate translation and implementation of EV‐based products.

## 4 CONCLUSIONS

During the “massivEVs” workshop, two full days were dedicated to the discussion of large‐scale EV production and manufacturing. Merging diverse competencies and experiences, spanning from academic research, clinics and industry, we reached the following points of consensus, summarized in Box 3.

**Box 3 ‐ Points of Consensus**

1. Prioritize function over purity. Potency comes first, then assessment of safety and whether purity issues influence both efficacy and/or safety.

2. A multi‐modal matrix of defined assays can help to establish QC of the EV product.

3.There is a great and unexploited potential in non‐human EV sources such as bacteria, non‐human milk, microalgae, etc.

4. Cross‐fertilization from different fields and interaction between academia and industry will be beneficial for all. A common language and guidelines would help in the conversation.

With respect to the point 4 of Box 3, it is worth to note that a lively discussion arose around the possibility/opportunity to also use the “exosome” term not only to indicate the Multivescicular Body (MVB)‐derived EVs but as a generic descriptor of EVs, by following a diffuse habit, especially in the industry. However, despite the popularity of the “exosome” term, the “EV” term has now been adopted by the international community as the consensus generic term for lipid bilayer‐delimited particles released from the cell, which includes exosomes (Théry et al., 2018; Witwer & Théry, 2019). The majority of the participants therefore agreed sticking to this nomenclature, also considering that having a unique definition is fundamental to improve communication and promote a common language.

According to the comments of the “massivEVs” participants, this workshop was a great success, not only from the scientific point of view, but also from the social side. The International Organizing Committee (IOC) and participants of the workshop were enthusiastically gratified by the opportunity to be the first to resume in‐person meeting opportunity. It gave all attendees in Desenzano a chance to taste and feel one of the most beautiful sides of the scientific world: gathering together and creating from the congress a forge of ideas, mutual enrichment, and the beginning of new, productive scientific liaisons.

### 4.1 MassivEVs website

https://www.isev.org/index.php?option=com_jevents&task=icalrepeat.detail&evid=1&Itemid=115&year=2021&month=10&day=28&title=massiveevs-workshop&uid=25bd26489811d8898c243c5e1f64e2de

## AUTHOR CONTRIBUTIONS

Lucia Paolini: Conceptualization; Funding acquisition; Supervision; Writing – original draft; Writing – review & editing. Marta Costa: Writing – original draft; Writing – review & editing. Fabio Antenucci: Writing – review & editing. Mario Barilani: Writing – review & editing. Marta Clos‐Sansalvador: Writing – review & editing. André Cronemberger Andrade: Writing – review & editing. Sara Giancaterino: Writing – review & editing. Stephanie M. Kronstadt: Writing – review & editing. Rachel R. Mizenko: Writing – review & editing. Muhammad Nawaz: Writing – review & editing. Xabier Osteikoetxea: Writing – review & editing. Carla Pereira: Writing – review & editing. Surya Shrivastava: Writing – review & editing. Anders Toftegaard Boysen: Writing – review & editing. Xiaoqin Wang: Writing – review & editing. Dionysios C. Watson: Writing – review & editing. Mario Gimona: Writing – review & editing. Maria Kaparakis‐Liaskos: Writing – review & editing. Konstantin Konstantinov: Writing – review & editing. Sai Kiang Lim: Writing – review & editing. Nicole Meisner‐Kober: Writing – review & editing. Michiel Stork: Writing – review & editing. Peter Nejsum: Conceptualization; Writing – review & editing. Annalisa Radeghieri: Conceptualization; Funding acquisition; Writing – review & editing. Eva Rohde: Conceptualization; Writing – original draft; Writing – review & editing. Nicolas Touzet: Conceptualization; Writing – review & editing. Kenneth W. Witwer: Conceptualization; Funding acquisition; Writing – review & editing. Antonella Bongiovanni: Conceptualization; Funding acquisition; Supervision; Writing – review & editing. Paolo BERGESE: Conceptualization; Funding acquisition; Supervision; Writing – review & editing.

## WORKSHOP IOC

Paolo Bergese (Co‐Chair); Antonella Bongiovanni (Co‐Chair); Peter Nejsum; Lucia Paolini; Annalisa Radeghieri; Eva Rohde; Nicolas Touzet; Marca H.M. Wauben; Kenneth W. Witwer.

## CONFLICTS OF INTEREST

No potential conflict of interest was reported by the authors.
